# RETRACTION: Comparative Analysis of Pressure Ulcer Development in Stroke Patients Within and Outside Healthcare Facilities: A Systematic Review and Meta‐Analysis

**DOI:** 10.1111/iwj.70545

**Published:** 2025-04-18

## Abstract

**RETRACTION:** YuG. , SunC. , HaoS. , and WuH. , “Comparative Analysis of Pressure Ulcer Development in Stroke Patients Within and Outside Healthcare Facilities: A Systematic Review and Meta‐Analysis,” International Wound Journal 21, no. 4 (2024): e14840, 10.1111/iwj.14840.38556516 PMC10982073

The above article, published online on 31 March 2024, in Wiley Online Library (http://onlinelibrary.wiley.com/), has been retracted by agreement between the journal Editor in Chief, Professor Keith Harding; and John Wiley & Sons Ltd. Following an investigation by the publisher, all parties have concluded that this article was accepted solely on the basis of a compromised peer review process. The editors have therefore decided to retract the article. The authors did not respond to our notice regarding the retraction.

**RETRACTION:** G. Yu , C. Sun , S. Hao , and H. Wu , “Comparative Analysis of Pressure Ulcer Development in Stroke Patients Within and Outside Healthcare Facilities: A Systematic Review and Meta‐Analysis,” International Wound Journal 21, no. 4 (2024): e14840, 10.1111/iwj.14840.38556516 PMC10982073

The above article, published online on 31 March 2024, in Wiley Online Library (http://onlinelibrary.wiley.com/), has been retracted by agreement between the journal Editor in Chief, Professor Keith Harding; and John Wiley & Sons Ltd. Following an investigation by the publisher, all parties have concluded that this article was accepted solely on the basis of a compromised peer review process. The editors have therefore decided to retract the article. The authors did not respond to our notice regarding the retraction.

